# Effects of Chestnut Tannin Extract, Vescalagin and Gallic Acid on the Dimethyl Acetals Profile and Microbial Community Composition in Rumen Liquor: An In Vitro Study

**DOI:** 10.3390/microorganisms7070202

**Published:** 2019-07-18

**Authors:** Federica Mannelli, Matteo Daghio, Susana P. Alves, Rui J. B. Bessa, Sara Minieri, Luciana Giovannetti, Giuseppe Conte, Marcello Mele, Anna Messini, Stefano Rapaccini, Carlo Viti, Arianna Buccioni

**Affiliations:** 1 Dipartimento di Scienze e Tecnologie Agrarie, Alimentari, Ambientali e Forestali, University of Florence, Piazzale delle Cascine 18, 50144 Firenze, Italy; 2 CIISA, Centro de Investigação Interdisciplinar em Sanidade Animal, Faculdade de Medicina Veterinária, Universidade de Lisboa, Avenida da Universidade Técnica, 1300-477 Lisboa, Portugal; 3 Dipartimento di Scienze Veterinarie, University of Pisa, Viale delle Piagge 2, 56124 Pisa, Italy; 4 Dipartimento di Scienze Agrarie, Alimentari e Agro-ambientali, University of Pisa, Via del Borghetto 80, 56124 Pisa, Italy

**Keywords:** chestnut tannin, vescalagin, gallic acid, rumen microbiota, dimethyl acetals, HTS-technology

## Abstract

The addition of polyphenol extracts in ruminant diets is an effective strategy to modulate rumen microflora. The aim of this in vitro trial was to study the effects of chestnut tannin extract (CHT), vescalagin (VES) and gallic acid (GAL) on dietary fibre degradability and on the dimethyl acetals (DMA) profile and microbial community composition of rumen liquor. Four diets (basal diet; basal diet plus CHT; basal diet plus VES; basal diet plus GAL) were fermented for 24 h using ewe rumen liquor. At the end of the fermentation, the microbial communities were characterized by sequencing the 16S rRNA gene. The DMA profile was analyzed by gas chromatography. Chestnut tannin extract did not affect fibre degradability, whereas VES and GAL showed a detrimental effect. The presence of CHT, VES and GAL influenced the concentration of several DMA (i.e., 12:0, 13:0, 14:0, 15:0, 18:0 and 18:1 *trans*-11), whereas the composition of the microbial community was marginally affected. The inclusion of CHT led to the enrichment of the genera *Anaerovibrio*, *Bibersteinia*, *Escherichia/Shigella*, *Pseudobutyrivibrio* and *Streptococcus*. The results of this study support the hypothesis that the activity of CHT is due to the synergistic effect of all components rather than the property of a single component.

## 1. Introduction

The Food and Agricultural Organization of United Nations reported that the increasing demand of protein with high biological value (e.g., from meat and milk) will lead to an expansion of livestock production, which, in turn, will lead to increasing environmental problems. In particular, ruminants are the main contributors of methane emissions in the atmosphere [1]. Rumen can be considered a fermenter in which many bacterial species cooperate for the transformation of the nutrients ingested by the host animal. During the fermentation of dietary fibre, hydrogen is produced. Its accumulation in rumen liquor can have toxic effects on the microbiota. Hydrogen is removed by the synthesis of methane that is emitted during animal eructation [2]. For this reason, several feeding strategies were developed to lower methane emissions and to improve the environmental sustainability of ruminant production systems [3].

Methane emissions from ruminants can be efficiently lowered by polyphenols, but the decrease of methanogenesis is often associated with a lower fibre degradability and, consequently, to a reduced production of acetate [4,5,6,7]. Chestnut tannins (CHT) were effective in decreasing methanogenesis without compromising acetate production [8,9]. Chestnut tannins are hydrolysable polyphenols composed by several fractions, including vescalagin (VES) and gallic acid (GAL), which account for nearly 20% and 6% of total tannins, respectively [10].

Data reported in literature showed that the use of CHT in ruminant feeding might result in changes in the profile of rumen microbial community [9,11,12]. Vescalagin is the main active component present in CHT [13], while GAL is quickly released in rumen liquor (RL) by hydrolysis of CHT [14]. These compounds were studied in medicine and veterinary science for their potential use as antimicrobials [15]. Indeed, Quideau et al. [13] studied several nonahydroxyterphenoyl-containing C-glycosidic ellagitannins (i.e., castalagin and VES from *Castanea* spp.) and found that VES was the most effective against both acyclovir-susceptible and acyclovir-resistant *herpes simplex virus* strains, showing a 50% inhibitory concentration (IC_50_) at 0.04 nM. Panizzi et al. [16] demonstrated the efficacy of GAL as antimicrobial against *Staphylococcus aureus*, *Bacillus cereus*, *Pseudomonas aeruginosa*, *Escherichia coli*, *Saccharomyces cerevisiae* and *Candida albicans*.

In this in vitro study, the effects of CHT, VES and GAL on neutral detergent fibre (NDF) degradability, microbial community profile and dimethyl acetals (DMA) composition of rumen liquor were evaluated, in order to assess the effect of the phytocomplex (i.e., CHT) versus the effect of the main individual components of CHT.

## 2. Material and Method

### 2.1. Diets

The control feed was formulated using tannin-free ingredients and was the same diet used in a previous in vivo trial in order to have a reference for animal performance and welfare [17]. The control diet (diet C) was composed by grass hay (609.76 g/kg dry matter—DM), barley (86.99 g/kg DM), maize meal (85.98 g/kg DM), wheat bran (64.53 g/kg DM), soybean meal (51.63 g/kg DM), beet pulp (36.59 g/kg DM), soybean oil (34.45 g/kg DM), molasses (17.28 g/kg on DM), mineral-vitamin supplement (12.80 g/kg DM). Three other diets were obtained by adding to diet C, either 16.0 g/kg DM of CHT extract (diet T) or 2.4 g/kg DM of VES (diet V) or 0.32 g/kg DM of GAL (diet G). Chestnut tannins, VES and GAL did not replace any ingredient of the diet C, but were simply added to diet C. Chestnut tannin powder was provided by Gruppo Mauro Saviola srl (plant in Radicofani, Siena, Italy) and contained 750 g equivalents of tannic acid/kg DM, determined according to the method of Burns [18]. Vescalagin and GAL were purchased by Sigma-Aldrich (cods 76418 and G7384, respectively; Sigma-Aldrich, St. Louis, MO). The inclusion level of VES and GAL in the diets was calculated after characterization of CHT extract according to the method reported by Bargiacchi et al. [10].

### 2.2. Proximate Analyses of Feed Samples

Samples of diet C were oven-dried at 60 °C for 24 h. Dry samples were analyzed for crude protein (CP), ether extract (EE) and ash according to AOAC methods (976.06, 920.39 and 942.05 procedures, respectively) [19]. Fibre fractions were determined according to van Soest et al. [20] as follows: NDF was determined using sodium sulphite and heat stable amylase; acid detergent fibre (ADF) and acid detergent lignin (ADL) were determined in a sequential analysis. All data were expressed inclusive of residual ash.

The chemical composition of the control diet was: DM, 920 g/kg of feeds; CP, 124 g/kg DM; EE, 48.7 g/kg DM; NDF, 466 g/kg DM, ADF, 333 g/kg DM; ADL, 64.3 g/kg DM. The concentrations of VES and GAL inclusion in the diets were confirmed according to Bargiacchi et al. [10], as previously reported.

### 2.3. Rumen Inoculum

The whole rumen content was collected from one ewe sacrificed at the slaughterhouse and used as inoculum for the fermentation, according to Denek et al. [21] and Lutakome et al. [22]. The animal was fed for one month before the slaughtering with the diet C used in the in vitro trial. The ewe was not sacrificed specifically for the experiment but slaughtered as a culling ewe.

The whole rumen was immediately transferred to the laboratory in a thermostatic box (39 °C). Then, RL was collected and filtered through four layers of cheese cloth into a flask under a flux of CO_2_, as described by Buccioni et al. [23]. Six hundred mL of rumen liquor were buffered (1:3, *v*/*v*) by adding an artificial saliva solution [24]. Feeds (1.0 g DM) were incubated (six fermenters per diet) with 100 mL of buffered inoculum and pH was monitored continuously during the fermentation [23]. After filtration and buffering, and before the fermentation, three samples of fresh RL (100 mL) were freeze-dried and analyzed as blanks to control the presence of possible artefacts. After 24 h of incubation samples were collected from each fermenter and, after measuring the pH, were stored at −80 °C. Three fermenters per diet were used for the determination of DMA profile and for the characterization of the microbial community; three fermenters per diet were used for the determination of NDF degradability (NDF_deg_), as described below.

### 2.4. DMA Analysis

Frozen samples were freeze-dried and DMA composition was determined according to Alves et al. [25], using C19:0 (1 mg/mL) as an internal standard. Briefly, freeze-dried rumen samples were trans-esterified using a combined basic followed by acid catalysis procedure. Then, DMA were isolated by thin-layer chromatography using dichloromethane as elution solvent. The DMA profile of all samples was then determined using a Shimadzu GC2010-Plus gas chromatograph (Shimadzu, Kyoto, Japan) equipped with a flame-ionization detector and a high polar fused-silica capillary column (SP-2560, 100 m × 0.25 mm, 0.20 μm film thickness, Supelco, Bellefont, PA, USA). Helium was used as the carrier gas at a flow of 1 mL/min. A split/splitless injector was used with a split ratio of 1:10. An aliquot of the sample was injected under the following GC conditions: the oven temperature started at 50 °C and held for 1 min; temperature was then increased to 150 °C at a rate of 50 °C/min, and held for 20 min, before being increased to 190 °C at 1 °C/min and then to 220 °C at a rate of 2 °C/min, and held for 40 min. The injector temperature was set at 220 °C and the detector temperature was set at 280 °C. Identification of DMA was achieved by electron impact mass spectrometry using a Shimadzu GC–MS QP2010 Plus (Shimadzu) and according to published chromatograms [25]. The GC-MS chromatographic column and the GC conditions were similar to the GC-FID analysis. Additional mass spectrometer conditions were as follows: ion source temperature, 200 °C; interface temperature, 240 °C; emission voltage, 70 eV. All DMA results were expressed as g/100 g of DM.

### 2.5. Rumen Degradability

Neutral detergent fibre degradability was determined according to Tilley and Terry [26] limiting the procedure only to the first step and not considering the degradability with pepsin. Neutral detergent fibre residuals (NDF_undeg_) were determined according to van Soest et al. [20]. Neutral detergent fibre degradability was calculated by difference between NDF of feeds (NDF_feed_), before the fermentation, and the NDF_undeg_, after 24 h of incubation:

NDF_deg_ = (NDF_feed_ − NDF_undeg_)/NDF_feed_ × 100 (1)

### 2.6. Quantification of the 16S rRNA Gene by Quantitative PCR

Abundance of total bacteria was estimated by quantification of the copy number of the 16S rRNA gene by quantitative PCR (qPCR) as previously reported [11] using universal primers to target partial 16S rRNA genes of total bacteria [27]. The analysis was performed using a CFX96 Real-Time PCR Detection System (Bio-Rad Laboratories, Hertfordshire, UK). Amplification conditions were 95 °C for 3 min, 40 cycles of 95 °C for 15 s and 60 °C for 30 s.

### 2.7. Amplification of 16S rRNA Gene, Sequencing and Sequence Analysis

DNA was extracted using the Fast DNA Spin kit for soil (MP Biomedicals, Solon, OH) according to Mannelli et al. [28]. DNA purity and quantity were measured using a ND-1000 Spectrophotometer (NanoDrop Technologies, Labtech, Ringmer, UK) and standardized to a concentration of 10 ng/μL. The V3-V4 hypervariable regions of the 16S rRNA gene were PCR-amplified using the Pro341f and Pro805R primers [29]. Sequencing was performed at BMR Genomics (Padova, Italy) by MiSeq Illumina (Illumina, Inc., San Diego, CA, USA) using a 300 bp × 2 paired end protocol. The sequencing produced a total of 1,004,351 reads with an average of 83,696 ± 3221 reads per sample (average ± standard error). Bioinformatic elaborations were performed in R 3.5.1 [30] with DADA2 package [31], version 1.8.0. The first 20 bases were removed from both forward and reverse reads, additionally forward reads were truncated at 280 bases and reverse reads were truncated at 250 bases. The reads with expected errors higher than 0.5 were discarded i.e., maxEE = 0.5 where EE = sum(10^−Q/10^). Specific error rates were estimated for the forward reads and for the reverse reads. Filtered reads were dereplicated, the estimated error rates were used to infer the amplicon sequence variants (ASVs) [32] and the read pairs were merged with default parameters. Chimeric sequences were removed. A total of 100,365 high-quality sequences were obtained with an average of 8,364 ± 490 sequences per sample (average ± standard error). Taxonomic assignment for each ASV was performed against the ribosomal database project (RDP) database [33] (confidence 80%). To estimate the alpha-diversity within each sample group, a randomly rarefied dataset (sample size = 4000 sequences) was generated, then, the Shannon index and the ASV richness were calculated using the vegan package [34] in R 3.5.1 [30].

### 2.8. Statistical Analysis

Data for DMA, NDF_deg_ and microbial relative abundance at the genus level (previously tested for normal distribution) were processed by the General Linear Model of SAS [35] using the following linear model with diet as fixed factor:

Y_i_ = μ + Diet_i_ + e_ij_ (2)

where Y is the observation, μ is the overall mean, Diet is the fixed effect of i^th^ diet (i = 1 to 4), and e_ij_ is the residual error.

Only one probability (*p* ≤ 0.05) was used to identify significant differences between means for NDF and DMA, while *p* < 0.1 was considered for the relative abundance of the microbial taxa.

Pairwise correlation among bacterial taxa and DMA composition was performed by SAS, 9.2 [35]. A non-metric multidimensional scaling (NMDS) and a permutational multivariate analysis of variance (PERMANOVA) based on Hellinger transformed genus relative abundance data, calculated removing the unclassified reads, were performed using the vegan package [34] in R 3.5.1 [30] using the metaMDS and the adonis2 functions, respectively.

## 3. Results

### 3.1. Rumen Degradability and DMA Profile

Dietary supplementation with CHT did not affect NDF_deg_ compared to diet C. Conversely, the degradability of both diets V and G decreased compared to the diet C of about 4 and 6 g/100 g of DM, respectively (Table 1).

The presence of the CHT extract, VES and GAL in the basal diet modified the concentration of several DMA in the RL (Table 2). After 24 h, the inclusion of CHT, GAL and VES increased the concentration of DMA 12:0, 13:0, 14:0, 15:0 *iso*, 16:1, 18:0 and 18:1 *trans*-11 compared to diet C (*p* ≤ 0.05). The administration of VES and GAL increased the concentration of DMA 14:0 *iso* compared to diet C. All treatments showed an increase in the concentration of DMA 17:0 and DMA 18:1 *cis*-9 and the highest values were reached with CHT (for DMA 17:0) and with CHT and VES (for DMA 18:1 *cis*-9).

### 3.2. Microbial Community Composition

The number of copies of the 16S rRNA gene per mL of RL was around 10^8^ for all treatments (Figure S1). The taxonomic composition of the microbial communities selected at the end of the in vitro trial was investigated by high-throughput sequencing (HTS) of the 16S rRNA gene. Amplicons sequence variants rarefaction analysis (Figure S2) indicated that the sequencing depth was enough to describe the biodiversity within the dataset. The Shannon index ranged between 4.2 ± 0.1 (diet G) and 4.30 ± 0.03 (diet C), while the ASVs richness ranged between 147 ± 16 (diet G) and 156 ± 3 (diet C) (Table 3). In total, 14 phyla, 22 classes, 24 orders, 31 families and 44 genera were identified within the whole dataset. More than 70% of the microorganisms classified at phylum level in each group were from the phyla *Proteobacteria* (from ~33% to ~38%) and *Bacteroidetes* (from ~35% to ~37%). The phylum *Proteobacteria* was represented mainly by *Gammaproteobacteria* of the families *Succinivibrionaceae*, *Moraxellaceae* and *Pasteurellaceae* (order *Aeromonadales*, *Pseudomonadales* and *Pasteurellales*, respectively) and, to a minor extent, by *Betaproteobacteria* of the family *Neisseriaceae* (order *Neisseriales*). The phylum *Bacteroidetes* was represented mainly by the family *Prevotellaceae* (order *Bacteroidales*, class *Bacteroidia*) (Figure S3 and Tables S1–S4). 

**Figure 1:**
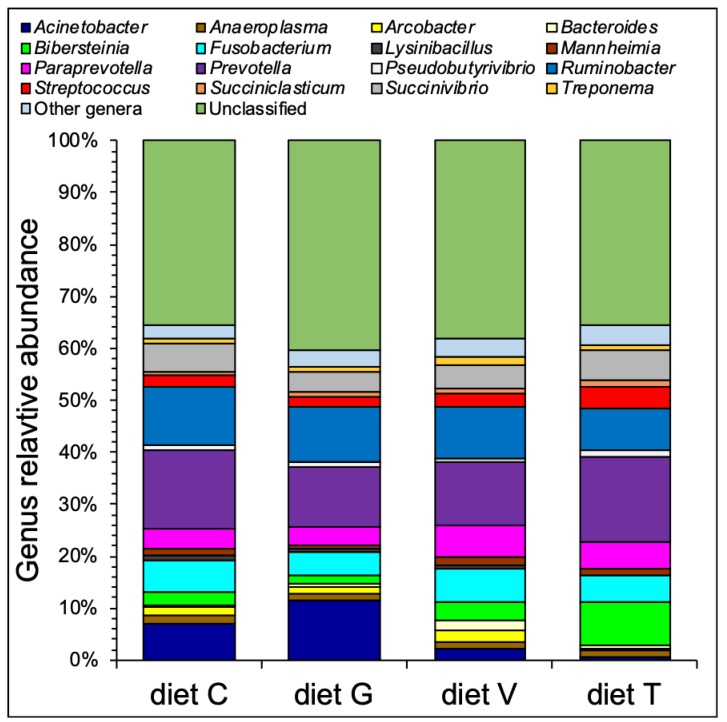
Taxonomic composition at genus level of the microbial communities enriched during the in vitro trial. Average abundances are reported for each tested condition. Only the genera with an average relative abundance of 1% (or higher) in at least one condition are reported.

A core microbiota (i.e., taxonomic groups shared by all conditions) was composed of 25 genera (Table 4). The most abundant genera (Figure 1 and Table 5) within the core microbiota were *Prevotella* (between ~11%—diet G, and ~16%—diet T), *Ruminobacter* (between ~8%—diet T, and ~11%—diet C) and *Fusobacterium* (between ~4%—diet G, and ~7%—diet V).

**Figure 2:**
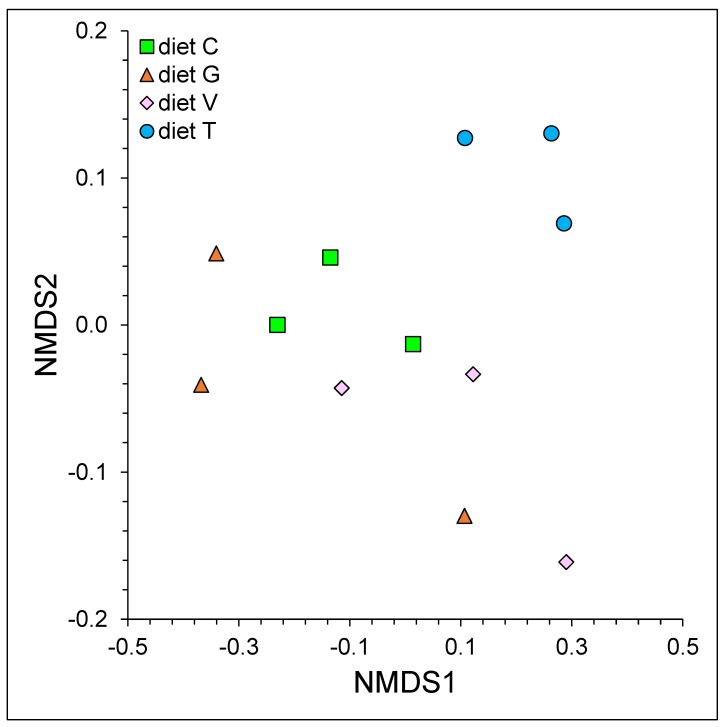
Non-metric multidimensional scaling (NMDS) based on Hellinger transformed genus relative abundance data.

The PERMANOVA analysis applied to the Hellinger transformed genus relative abundance data indicated a difference between the microbial communities across the treatments (*p* ≤ 0.01). This result was confirmed by the NMDS plot (Figure 2): the samples from diet T clustered separately from the samples from the other treatments. Seven genera (*Anaerovibrio*, *Arcobacter*, *Bibersteinia*, *Escherichia/Shigella*, *Pseudobutyrivibrio*, *Streptococcus*, *Treponema*) of the 44 genera detected within the dataset showed significant differences in the relative abundance across treatments (Table 5). The post-hoc comparisons of the average genus relative abundance clearly showed a significant effect of diet T in shaping the microbial communities (Table 5). The genera *Anaerovibrio*, *Bibersteinia*, *Escherichia/Shigella* and *Streptococcus* were enriched in the fermenters containing diet T, compared to the other treatments. The relative abundance of the genus *Pseudobutyrivibrio* increased with diet T compared to diet C and diet V. Conversely, the relative abundance of the genus *Arcobacter* decreased with diet T. Furthermore, a slight decrease in the relative abundance of the genus *Treponema* was observed in the fermenters with diet G compared to the fermenters with diet V.

In addition to the taxonomic composition, the presence of CHT or VES and GAL strongly influenced the correlations between the identified DMA and the microorganisms (Table S5). Only the genus *Bibersteinia* showed a negative correlation to DMA 18:1 *trans*-11 in two different diets (i.e., diet C and diet V).

## 4. Discussion

The inclusion of polyphenols in ruminant diets can modulate the diversity and activity of rumen microorganisms, the nutrient degradability and rumen methanogenesis [36]. In the present trial, the inclusion of CHT did not affect the degradability of dietary NDF compared to the control. The low value of NDF_deg_ in all fermenters suggested that the conditions of in vitro trials were not optimal for the evaluation of fibre degradability. As a consequence, data related to NDF_deg_ have to be considered only as qualitative markers (i.e., referred to the control). Several authors reported an increase in acetic acid concentration in RL from dairy ewes fed diets supplemented with 10–30 g/kg DM intake (DMI) [8] or 16 g/kg DMI [9] of CHT extract, demonstrating that this kind of polyphenol had no detrimental effect on the activity of cellulolytic bacteria. In contrast, Zimmer and Cordesse [37] found that CHT decreased in vivo apparent organic matter (OM) digestibility in ewes and goats. Tabacco et al. [38] confirmed that lucerne ensiled with CHT reduced in vitro OM digestibility by 5%. The percentage of tannins included in the diet and the kind of polyphenol certainly play an important role and could explain the inconsistency of many data reported in the literature. In the present experiment, the CHT inclusion was about 1.6 g/100 g of DM, lower than in the trials cited above.

The ability of polyphenols in lowering methane emissions is usually a consequence of a decreasing NDF degradation, which, in turn, leads to a reduction of acetate synthesis and, eventually, to a decrease in the availability of electron donors for the methanogens [2]. Hence, the reduction in methane production is often linked to a decrease in NDF rumen degradability. The findings of the present study confirmed that CHT had no detrimental effects on NDF_deg_ and the activity of cellulolytic bacteria. Since several authors reported that CHT dietary supplementation was been associated with a lowering in methane production [7,8], the administration of CHT in ruminant feeding could be an interesting perspective to increase the environmental sustainability of livestock farming without affecting feed efficiency.

Tannins can modulate the rumen microbiota, inhibit microbial enzymes or complex cell wall components [39]. Commonly, condensed tannins decrease the rumen degradation of fibre because they induce important changes in the microbial community, while the effect of hydrolysable tannins is usually milder [40]. Chestnut tannin extract has been reported to be less effective on the growth of cellulolytic bacteria than condensed tannins, such as quebracho or mimosa [11,12,40,41]. A plausible explanation for this behaviour could be related to the higher grade of depolymerization of hydrolysable tannins in rumen compared to condensed tannins [42]. Recently, in an in vivo trial with fistulated sheep, Costa et al. [40] compared mimosa tannins and CHT. The authors observed a reduced growth of cellulolytic bacteria with condensed tannins compared to hydrolysable tannins. Moreover, Liu et al. [8] found that in vitro acetate production was not affected by CHT, confirming that this kind of tannin extract did not influence the activity of cellulolytic bacteria. This result is consistent with our microbiological findings that show only marginal changes in the composition of the microbial community.

To better understand the role of single components of CHT, the effect of VES and GAL on NDF degradability was evaluated. Vescalagin and GAL reduced NDF degradability. However, no considerable changes in the composition of the microbial community were observed compared to diet C. These findings can be explained considering the possibility that VES and GAL could i) inhibit the activity of the microorganisms; ii) inhibit the enzymes involved in NDF degradation; or iii) complex the fibre, thus making it unavailable [43,44]. Since the microbial community was characterized by DNA extraction and HTS of the 16S rRNA gene, it is not possible to discern between active and inactive microorganisms in the present trial. Indeed, a recent comparison of the results obtained by RNA amplicon sequencing and DNA amplicon sequencing showed a different relative abundance of the main bacterial phyla such as *Bacteroidetes* (22.7 ± 8.1% and 50.3 ±8.7%, respectively) and *Proteobacteria* (46.3 ± 14.3% and 4.3 ± 8.5%, respectively) [45].

To improve knowledge on the effects of CHT, VES and GAL on rumen microbial communities, the DMA profile was investigated, and a metataxonomic approach was exploited.

Dimethyl acetals are derived from the plasmalogen lipids of bacterial membranes, and their composition is similar to fatty acid profile showing odd, even, saturated and unsaturated chains from C12 to C18. Their variation is strongly linked to the ability of bacteria to be resilient to environmental changes, modifying their membrane fluidity as a defence strategy [46,47]. Alves et al. [25] showed that DMA could be an efficient tool as microbial condition marker.

In our trial, DMA 15:0 *iso* increased and it could be symptomatic of adaptation of cellulolytic bacteria to the stimuli induced by CHT, VES, and GAL present in RL. Similar considerations may be applied to DMA 17:0 *ante* for amylolytic bacteria. Our data are consistent with the findings reported by Costa et al. [40], since the inclusion of CHT, or of its components, in the diet was able to affect the concentration of DMA 13:0, 14:0 *iso*, 16:1 and 18:0. Moreover, the presence of DMA 18:1 *trans*-11 may be considered a marker of the incorporation in the structural lipids of the biohydrogenation intermediates, as previously reported also by Alves et al. [25].

Our study showed diet-specific correlations among several plasmalogen derivatives and microbial genera, confirming the importance of DMA composition as a tool of microbial characterization in a specific environmental condition.

Metataxonomic analysis of RL inoculated with CHT, VES, and GAL showed that only the genera *Prevotella*, *Paraprevotella*, *Succinivibrio,* and *Treponema* were detected in all conditions. In a previous study, Henderson et al. [48] described a “core bacterial microbiome”, that was composed by key microorganisms detected in all samples: *Prevotella*, *Butyrivibrio*, *Ruminococcus*, unclassified members of the families *Lachnospiraceae* and *Ruminococcaceae*, as well as unclassified *Bacteroidales* and *Clostridiales*. In another study, the genera *Paraprevotella*, *Succinivibrio*, *Treponema*, *Fibrobacter,* and *Oscillibacter*, in addition to the above mentioned *Prevotella*, *Butyrivibrio* and *Ruminococcus* were identified as core genera in rumen bacterial communities of pre-ruminant dairy calves, cows and beef steers [49]. The comparison of our results with the core microbiome described in previous studies [48,49] highlighted the importance of the genus *Prevotella* in the rumen’s ecology since this genus is involved in the utilization of hemicellulose [49,50]. Moreover, in our study, members of the genus *Oscillibacter* were not detected in the whole dataset. Since changes in the detected microorganisms in different studies can be due to the use of different primer sets, to exclude this hypothesis, the 16S rRNA gene sequences of different strains of *Oscillibacter* were retrieved from GenBank [51] (accession numbers NR_118156.1, HM626173.1, NR_074793.2) and aligned to the primer sequences. The alignment confirmed that our primer set was able to target also the genus *Oscillibacter*. A possible reason of the differences observed between this in vitro study and the core rumen communities studied in vivo by Henderson et al. [48] and by Wu et al. [49] can be the absence of microorganisms-host interactions (e.g., absence of immune system) that characterized the in vitro trials. However, the overall behaviour can be considered reliable since in vitro experiments are widely used [7,23,52].

Regarding the microbial genera affected by CHT, VES, and GAL (i.e., *Anaerovibrio*, *Arcobacter*, *Bibersteinia*, *Escherichia/Shigella*, *Pseudobutyrivibrio*, *Streptococcus*, *Treponema*), little information is available in literature. The relative abundance of *Streptococcus* spp. increased in diet T, in accordance with Costa et al. [40], who showed a higher abundance of this genus in RL from ewes fed CHT compared to RL from ewes fed mimosa tannins or a mix of CHT extract and mimosa. Furthermore, our metataxonomic data showed that diet T increased the relative abundance of the genus *Anaerovibrio,* which was negatively affected by polyphenols from olive oil pomace in a previous study [28].

The reason for the different responses of bacteria to tannins is unclear, and factors such as dose, type of tannin and purity of the extract contribute to explain the conflicting results reported in the literature.

## 5. Conclusions

At the inclusion level used in this trial, CHT, VES and GAL did not show detrimental effects on the rumen’s microbial community. A correlation among several plasmalogens and microbial genera was found, confirming the importance of DMA composition as a tool in understanding the potential effect of dietary changes on the rumen’s microbial community. Moreover, the activity of CHT is likely due to its complex structure, rather than its single components (e.g., VES or GAL). In this in vitro trial, the presence of CHT did not decrease NDF_deg_ compared to the control, suggesting that the administration of CHT in ruminant feeding could be a useful strategy to reduce methanogenesis, without affecting diet efficiency and animal performance. More studies are needed to investigate the interaction of tannins with rumen microbial communities to increase the synthesis of bioactive compounds at the rumen level.

## Figures and Tables

**Table 1:** Neutral detergent fibre (NDF) degradability among feeds.

	diet C	diet T	diet G	diet V	SEM ^1^	*p* ^2^
NDF g/100g DM	41.73 b	41.88 b	44.79 a	43.71 a	0.75	0.0380 *
Degradability g/100g DM	10.49 a	10.17 a	3.94 c	6.25 b	1.61	0.0380 *

^1^ Standard Error Mean. ^2^ Probability of significant effect due to experimental diets (* *p* ≤ 0.05); means within a row with different letters (a–c) are different (*p* ≤ 0.05).

**Table 2:** Profile of identified dimethyl acetals (DMA) in rumen liquor at 24 h (g/100g dry matter (DM)).

DMA	diet C	diet T	diet G	diet V	SEM ^1^	*p* ^2^
DMA 12:0	0.052 ^b^	0.257 ^a^	0.266 ^a^	0.234 ^a^	0.044	0.0294 *
DMA 13:0 *iso*	0.025	0.124	0.103	0.095	0.024	0.0868
DMA 13:0	0.044 ^b^	0.128 ^a^	0.124 ^a^	0.132 ^a^	0.020	0.0458 *
DMA 14:0 *iso*	0.365 ^b^	0.244 ^b^	1.318 ^a^	1.132 ^a^	0.152	0.0075 *
DMA 14:0	0.281 ^b^	0.824 ^a^	0.921 ^a^	0.800 ^a^	0.122	0.0238 *
DMA 15:0 *iso*	0.257 ^b^	0.652 ^a^	0.816 ^a^	0.700 ^a^	0.110	0.0329 *
DMA 15:0	0.280	0.593	0.477	0.530	0.223	0.7784
DMA 16:0	1.957	5.225	5.628	5.454	0.956	0.0767
DMA 16:1	0.074 ^c^	0.316 ^a^	0.212 ^b^	0.190 ^b^	0.035	0.0088 *
DMA 17:0 *iso*	0.041	0.122	0.145	0.144	0.029	0.1061
DMA 17:0 *ante*	0.054 ^c^	0.212 ^a^	0.214 ^a^	0.168 ^b^	0.024	0.0054 *
DMA 17:0	0.030 ^d^	0.181 ^a^	0.114 ^b^	0.083 ^c^	0.025	0.0200 *
DMA 18:0	0.186 ^b^	0.518 ^a^	0.572 ^a^	0.567 ^a^	0.093	0.0544
DMA 18:1 *trans*-11	0.030 ^b^	0.122 ^a^	0.087 ^a^	0.094 ^a^	0.015	0.0208 *
DMA 18:1 *cis*-9	0.173 ^b^	0.571 ^a^	0.408 ^b^	0.417 ^a,b^	0.077	0.0389 *
DMA 18:1 *cis*-11	0.087	0.294	0.166	0.227	0.048	0.0751
DMA 18:1 *cis*-12	0.024	0.081	0.026	0.040	0.020	0.2493
DMA 17:1	0.097	0.360	0.276	0.247	0.098	0.3558
DMA 18:2	0.099	0.904	0.250	0.185	0.284	0.2489
DMA 26:0	0.552	1.3700	0.961	1.3570	0.290	0.2283

^1^ Standard error mean. ^2^ Probability of significant effect due to experimental diets (* = *p* ≤ 0.05); means within a row with different letters (a, b, c) are different (*p* ≤ 0.05).

**Table 3:** Amplicon sequence variants (ASVs) number and Shannon Index calculated for each tested condition. Values are reported as average ± standard error.

	diet C	diet T	diet G	diet V
ASVs	156 ± 3	154 ± 12	147 ± 16	148 ± 2
Shannon Index	4.30 ± 0.03	4.3 ± 0.1	4.2 ± 0.1	4.29 ± 0.06

**Table 4:** Taxonomic groups shared by all conditions.

Phylum	Class	Order	Family	Genus
*Bacteroidetes*	*Bacilli*	*Aeromonadales*	*Acidaminococcaceae*	*Acinetobacter*
*Candidatus_Saccharibacteria*	*Bacteroidia*	*Anaeroplasmatales*	*Anaeroplasmataceae*	*Alloprevotella*
*Euryarchaeota*	*Betaproteobacteria*	*Bacteroidales*	*Bacteroidaceae*	*Anaeroplasma*
*Firmicutes*	*Clostridia*	*Burkholderiales*	*Campylobacteraceae*	*Anaerovibrio*
*Fusobacteria*	*Deltaproteobacteria*	*Campylobacterales*	*Clostridiales_Incertae_Sedis_XIII*	*Arcobacter*
*Proteobacteria*	*Epsilonproteobacteria*	*Clostridiales*	*Desulfovibrionaceae*	*Bacteroides*
*Spirochaetes*	*Erysipelotrichia*	*Desulfovibrionales*	*Erysipelotrichaceae*	*Bibersteinia*
*SR1*	*Fusobacteriia*	*Erysipelotrichales*	*Fusobacteriaceae*	*Campylobacter*
*Tenericutes*	*Gammaproteobacteria*	*Fusobacteriales*	*Lachnospiraceae*	*Desulfovibrio*
*Verrucomicrobia*	*Methanobacteria*	*Lactobacillales*	*Methanobacteriaceae*	*Fusobacterium*
	*Mollicutes*	*Methanobacteriales*	*Methanomassiliicoccaceae*	*Mannheimia*
	*Negativicutes*	*Methanomassiliicoccales*	*Moraxellaceae*	*Methanobrevibacter*
	*Spirochaetia*	*Neisseriales*	*Neisseriaceae*	*Methanomassiliicoccus*
	*Subdivision5*	*Pasteurellales*	*Pasteurellaceae*	*Moraxella*
	*Thermoplasmata*	*Pseudomonadales*	*Porphyromonadaceae*	*Paraprevotella*
		*Selenomonadales*	*Prevotellaceae*	*Prevotella*
		*Spirochaetales*	*Ruminococcaceae*	*Pseudobutyrivibrio*
			*Spirochaetaceae*	*Roseburia*
			*Streptococcaceae*	*Ruminobacter*
			*Succinivibrionaceae*	*Saccharofermentans*
			*Veillonellaceae*	*Selenomonas*
				*Streptococcus*
				*Succiniclasticum*
				*Succinivibrio*
				*Treponema*

**Table 5:** Relative abundance of the microorganisms classified at genus level (confidence 80%).

Domain	Phylum	Class	Order	Family	Genus	diet C (%)	diet T (%)	diet G (%)	diet V (%)	SEM ^1^	*p* ^2^
*Archaea*	*Euryarchaeota*	*Methanobacteria*	*Methanobacteriales*	*Methanobacteriaceae*	*Methanobrevibacter*	0.34	0.37	0.20	0.48	0.15	0.6309
					*Methanosphaera*	<0.01	<0.01	0.03	<0.01	0.01	0.4411
		*Thermoplasmata*	*Methanomassiliicoccales*	*Methanomassiliicoccaceae*	*Methanomassiliicoccus*	0.19	0.29	0.54	0.48	0.13	0.2790
*Bacteria*	*Bacteroidetes*	*Bacteroidia*	*Bacteroidales*	*Bacteroidaceae*	*Bacteroides*	0.28	0.57	0.38	1.96	0.48	0.1218
				*Porphyromonadaceae*	*Petrimonas*	0.03	N.D. ^3^	N.D.	0.04	0.03	0.5880
					*Porphyromonas*	<0.01	<0.01	<0.01	0.02	0.01	0.4411
				*Prevotellaceae*	*Alloprevotella*	0.04	0.05	0.07	0.09	0.03	0.6864
					*Paraprevotella*	3.81	5.25	3.67	6.16	0.91	0.2399
					*Prevotella*	15.15	16.16	11.46	11.97	1.53	0.1528
	*Elusimicrobia*	*Elusimicrobia*	*Elusimicrobiales*	*Elusimicrobiaceae*	*Elusimicrobium*	0.01	N.D.	0.01	N.D.	0.01	0.5948
	*Fibrobacteres*	*Fibrobacteria*	*Fibrobacterales*	*Fibrobacteraceae*	*Fibrobacter*	0.02	0.01	0.03	<0.01	0.02	0.7279
	*Firmicutes*	*Bacilli*	*Bacillales*	*Planococcaceae*	*Kurthia*	N.D.	N.D.	0.18	N.D.	0.09	0.4411
					*Lysinibacillus*	1.16	N.D.	0.69	0.67	0.39	0.2896
			*Lactobacillales*	*Streptococcaceae*	*Streptococcus*	2.19 ^b^	4.22 ^a^	1.99 ^b^	2.51 ^b^	0.24	0.0006 *
		*Clostridia*	*Clostridiales*	*Lachnospiraceae*	*Butyrivibrio*	<0.01	0.17	<0.01	<0.01	0.09	0.4411
					*Clostridium_XlVa*	N.D.	0.13	0.09	0.12	0.09	0.7078
					*Clostridium_XlVb*	N.D.	N.D.	N.D.	0.03	0.01	0.4411
					*Pseudobutyrivibrio*	0.71 ^b^	1.31 ^a^	0.99 ^a,b^	0.68 ^b^	0.10	0.0086 *
					*Roseburia*	0.21	0.14	0.22	0.22	0.10	0.9232
				*Peptostreptococcaceae*	*Peptostreptococcus*	<0.01	<0.01	<0.01	0.02	0.01	0.4411
				*Ruminococcaceae*	*Anaerofilum*	N.D.	N.D.	N.D.	0.03	0.01	0.4411
					*Clostridium_IV*	0.06	0.02	N.D.	0.06	0.03	0.5830
					*Ruminococcus*	0.14	0.03	0.10	<0.01	0.06	0.4272
					*Saccharofermentans*	0.04	0.11	0.11	0.06	0.06	0.7622
		*Negativicutes*	*Selenomonadales*	*Acidaminococcaceae*	*Succiniclasticum*	0.78	1.15	0.8	1.06	0.18	0.4620
				*Veillonellaceae*	*Anaerovibrio*	0.12 ^b^	0.53 ^a^	0.11 ^b^	0.15 ^b^	0.09	0.0317 *
					*Selenomonas*	0.58	0.94	0.47	0.56	0.12	0.1015
	*Fusobacteria*	*Fusobacteriia*	*Fusobacteriales*	*Fusobacteriaceae*	*Fusobacterium*	6.07	5.28	4.20	6.56	0.69	0.1647
	*Proteobacteria*	*Betaproteobacteria*	*Burkholderiales*	*Comamonadaceae*	*Brachymonas*	N.D.	0.03	N.D.	N.D.	0.01	0.4411
					*Comamonas*	N.D.	0.08	N.D.	N.D.	0.04	0.4411
		*Deltaproteobacteria*	*Desulfovibrionales*	*Desulfovibrionaceae*	*Desulfovibrio*	0.05	0.02	0.05	0.03	0.04	0.8945
		*Epsilonproteobacteria*	*Campylobacterales*	*Campylobacteraceae*	*Arcobacter*	1.84 ^a^	0.29 ^b^	1.39 ^a^	2.23 ^a^	0.25	0.0028 *
					*Campylobacter*	0.42	0.24	0.50	0.51	0.12	0.3658
		*Gammaproteobacteria*	*Aeromonadales*	*Succinivibrionaceae*	*Ruminobacter*	11.28	8.12	10.59	9.91	1.36	0.4438
					*Succinivibrio*	5.41	5.76	4.00	4.59	0.51	0.1443
			*Enterobacteriales*	*Enterobacteriaceae*	*Escherichia/Shigella*	0.02 ^b^	0.24 ^a^	N.D. ^b^	0.07 ^b^	0.03	0.0009 *
			*Pasteurellales*	*Pasteurellaceae*	*Actinobacillus*	0.06	0.14	N.D.	<0.01	0.04	0.1154
					*Bibersteinia*	2.39 ^b^	8.30 ^a^	1.86 ^b^	3.49 ^b^	0.87	0.0030 *
					*Mannheimia*	1.33	1.15	0.72	1.55	0.41	0.5536
			*Pseudomonadales*	*Moraxellaceae*	*Acinetobacter*	7.08	0.62	11.49	2.33	3.36	0.1752
					*Moraxella*	0.17	0.13	0.21	0.18	0.09	0.9269
	*Spirochaetes*	*Spirochaetia*	*Spirochaetales*	*Spirochaetaceae*	*Treponema*	1.04 ^a,b^	1.19 ^a,b^	0.82 ^b^	1.56 ^a^	0.17	0.0778 *
	*Synergistetes*	*Synergistia*	*Synergistales*	*Synergistaceae*	*Pyramidobacter*	<0.01	0.13	0.22	0.19	0.07	0.2345
	*Tenericutes*	*Mollicutes*	*Anaeroplasmatales*	*Anaeroplasmataceae*	*Anaeroplasma*	1.40	1.37	1.33	1.16	0.19	0.8156

^1^ Standard error of the mean. ^2^ Probability of significant effect due to experimental diets (* = *p* < 0.1). ^3^ N.D. = not detected (i.e., relative abundance = 0). Significant different relative abundances (*p* < 0.1) are reported with letters (a, b). For each genus the taxonomic classification is reported. The sum of the relative abundances for each sample is lower than 100% because unclassified sequences are not included in the table.

## Supplementary Materials

Supplementary materials can be found at https://www.mdpi.com/2076-2607/7/7/202/s1.

## Abbreviations

ADF	acid detergent fibre
ADL	acid detergent lignin
ASV	amplicon sequence variant
C	control
CHT	chestnut tannins
CP	crude protein
DM	dry matter
DMA	dimethyl acetals
DMI	DM intake
EE	ether extract
GAL	gallic acid
HTS	high-throughput sequencing
NDF	neutral detergent fibre
NDF_deg_	NDF degradability
NDF_feed_	NDF of feeds
NDF_undeg_	NDF residuals
OM	organic matter
qPCR	quantitative PCR
RL	rumen liquor
VES	vescalagin
