# Supplementary material for: Effects of Chestnut Tannin Extract, Vescalagin and Gallic Acid on the Dimethyl Acetals Profile and Microbial Community Composition in Rumen Liquor: An In Vitro Study
Source: Microorganisms. 2019 Jul 18;7(7):202. doi: 10.3390/microorganisms7070202 (PMC6680752; doi:10.3390/microorganisms7070202)
Supplement: Supplementary file 1 [file microorganisms-07-00202-s001.zip › Table S5.docx]

Table S5. Correlation between DMA and bacterial genus of interest (only correlation for P ≤ 0.05 are reported).

| DMA | diet C | C^1^ | diet T | C^1^ | diet G | C^1^ | diet V | C^1^ |
| --- | --- | --- | --- | --- | --- | --- | --- | --- |
| DMA-12:0 |  |  | *Bibersteinia* | + | *Alloprevotella* | + | *Lysinibacillus* | + |
|  |  |  | *Mannheimia* | + | *Prevotella* | + | *Ruminobacter* | + |
|  |  |  |  |  |  |  | *Streptococcus* | - |
| DMA-13:0 | *Acinetobacter* | + | *Selenomonas* | - | *Paraprevotella* | - |  |  |
|  | *Succiniclasticum* | - |  |  |  |  |  |  |
| DMA-14:0 | *Alloprevotella* | + | *Desulfovibrio* | - | *Succiniclasticum* | + | *Bibersteinia* | - |
|  |  |  | *Escherichia/Shigella* | - | *Treponema* | + | *Mannheimia* | - |
| DMA-16:1 | *Fusobacterium* | + | *Pseudobutyrivibrio* | - | *Succiniclasticum* | + | *Bacteroides* | - |
|  | *Selenomonas* | - |  |  | *Treponema* | + | *Fusobacterium* | - |
| DMA-17:0 | *Methanomassiliicoccus* | + |  |  | *Fusobacterium* | + | *Fusobacterium* | - |
|  |  |  |  |  | *Mannheimia* | + |  |  |
|  |  |  |  |  | *Paraprevotella* | - |  |  |
| DMA-18:0 | *Methanomassiliicoccus* | - |  |  |  |  | *Anaerovibrio* | + |
|  | *Pseudobutyrivibrio* | + |  |  |  |  | *Campylobacter* | - |
|  |  |  |  |  |  |  | *Desulfovibrio* | - |
|  |  |  |  |  |  |  | *Saccharofermentans* | - |
| DMA-18:1c9 | *Succiniclasticum* | - |  |  | *Streptococcus* | + | *Methanomassiliicoccus* | + |
|  |  |  |  |  |  |  | *Succinivibrio* | + |
| DMA-18:1t | *Bibersteinia* | - |  |  |  |  | *Bibersteinia* | - |
|  | *Saccharofermentans* | + |  |  |  |  | *Mannheimia* | - |
| DMA-18:2 |  |  |  |  | *Pseudobutyrivibrio* | + | *Escherichia/Shigella* | + |
|  |  |  |  |  |  |  | *Lysinibacillus* | + |
|  |  |  |  |  |  |  | *Pyramidobacter* | - |
|  |  |  |  |  |  |  | *Streptococcus* | - |
| DMA-a17:0 | *Anaerovibrio* | - | *Alloprevotella* | - |  |  | *Methanomassiliicoccus* | + |
|  | *Bibersteinia* | - |  |  |  |  | *Succinivibrio* | + |
|  | *Saccharofermentans* | + |  |  |  |  |  |  |
| DMA-i14:0 |  |  | *Desulfovibrio* | - | *Succiniclasticum* | + | *Bibersteinia* | - |
|  |  |  |  |  | *Treponema* | + | *Mannheimia* | - |
| DMA-i15:0 |  |  | *Desulfovibrio* | - |  |  | *Fusobacterium* | - |
|  |  |  | *Escherichia/Shigella* | - |  |  |  |  |

^1^Correlation: +, positive; -, negative.
