# Supplementary material for: Effects of Chestnut Tannin Extract, Vescalagin and Gallic Acid on the Dimethyl Acetals Profile and Microbial Community Composition in Rumen Liquor: An In Vitro Study
Source: Microorganisms. 2019 Jul 18;7(7):202. doi: 10.3390/microorganisms7070202 (PMC6680752; doi:10.3390/microorganisms7070202)
Supplement: Supplementary file 1 [file microorganisms-07-00202-s001.zip › Supplementary figures.docx]

Figure S1 – Results of the quantitative PCR on the 16S rRNA gene.


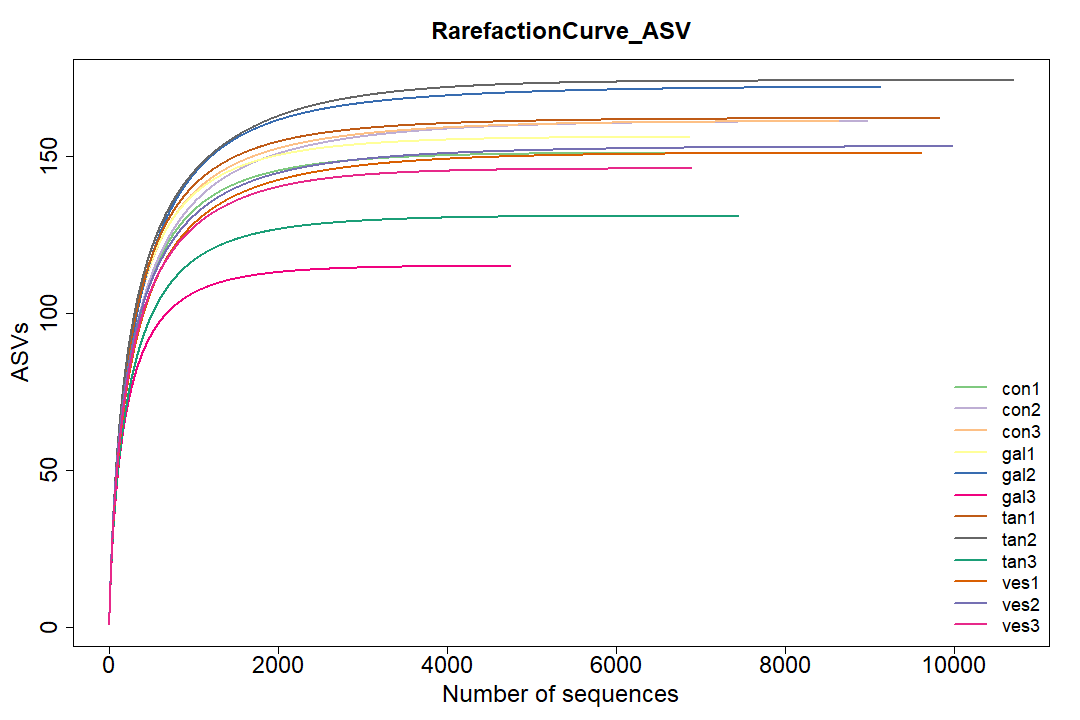


Figure S2 – Rarefaction curve calculated at genus level.


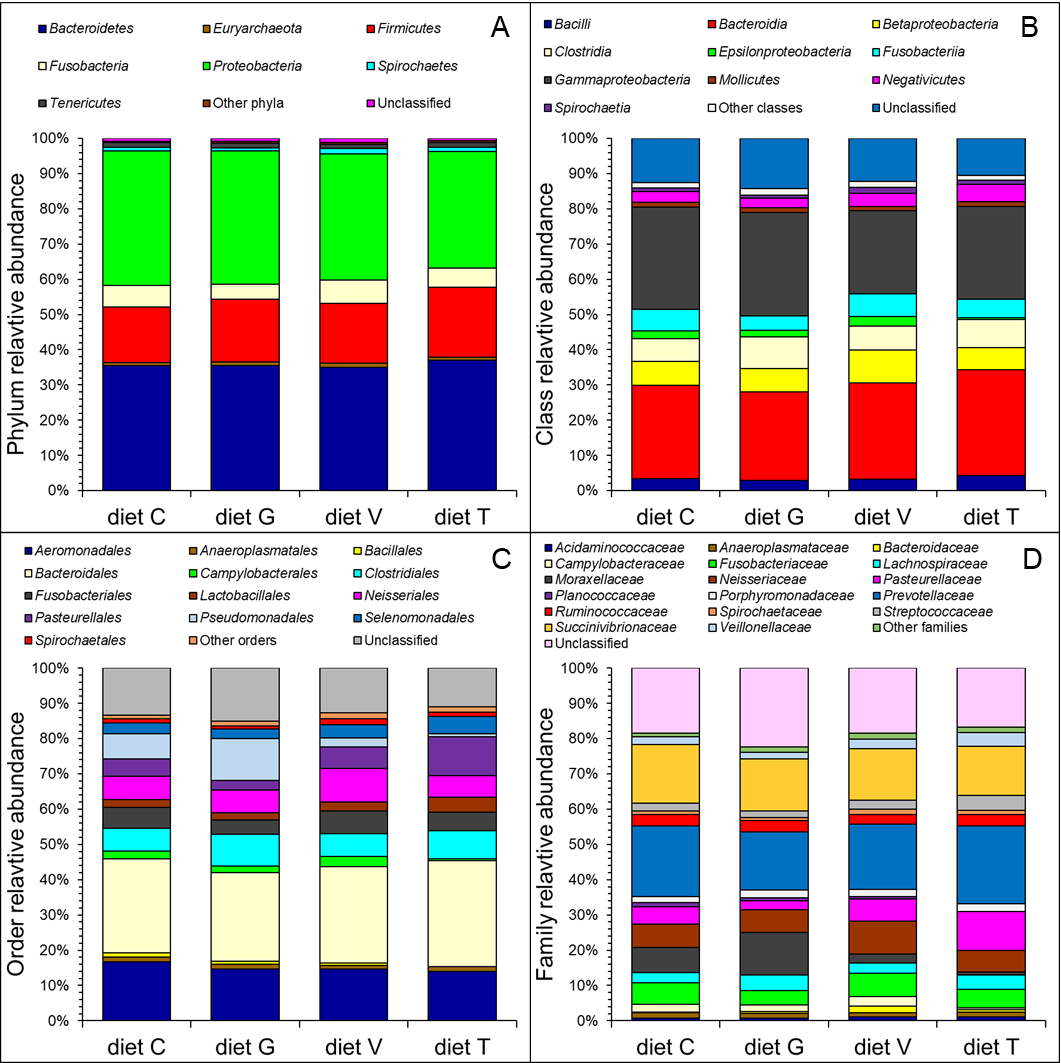


Figure S3 – Taxonomic composition at phylum (A), class (B), order (C) and family (D) level of the microbial communities enriched during the *in vitro* trial. Average abundances are reported for each tested condition. Only the groups with an average relative abundance of 1% (or higher) in at least one condition are reported.
